# Bearing the burden: Podoconiosis and mental health—A three-way comparative cross-sectional study in Rwanda

**DOI:** 10.1371/journal.pntd.0012346

**Published:** 2024-08-08

**Authors:** Natnael Shimelash, Theogene Uwizeyimana, Leila Dusabe, Jeanne Uwizeyimana, Tonya Huston, Janna M. Schurer

**Affiliations:** 1 Innovation Center, University of Global Health Equity, Butaro, Rwanda; 2 Bill & Joyce Cummings Institute of Global Health, University of Global Health Equity, Butaro, Rwanda; 3 Center for One Health, University of Global Health Equity, Butaro, Rwanda; 4 Heart and Sole Africa, Ruhengeri, Rwanda; 5 Hill Country Memorial Physician Practice, Fredericksburg, Texas, United States of America; 6 Department of Infectious Disease and Global Health, Cummings School of Veterinary Medicine at Tufts University, North Grafton, Massachusetts, United States of America; University of Sussex, UNITED KINGDOM OF GREAT BRITAIN AND NORTHERN IRELAND

## Abstract

Podoconiosis is a non-infectious, neglected tropical disease caused by chronic barefoot contact with irritant volcanic soils. It typically presents with lower limb swelling, disfigurement, and chronic disability. Patients and their families experience stigma from their communities. Depression, anxiety, and emotional distress contribute to the total illness burden of podoconiosis. This study used a survey-based comparative cross-sectional quantitative study design involving podoconiosis patients, their family members, and unaffected neighbors. The Depression, Anxiety, and Stress Scale (DASS 21), the WHO Quality of Life Scale (WHO-QOL Brief), and the Tekola clinical staging system were used to collect data. We surveyed 741 participants (33.1% patients, 33.3% family, 33.5% neighbors). Podoconiosis patients exhibited significantly elevated odds of severe depression (19.8x), anxiety (10.7x), and stress symptoms (13.5x) in comparison to unaffected neighbors. Family members of podoconiosis patients displayed 1.5x higher odds of experiencing severe anxiety symptoms compared to unaffected neighbors. Higher clinical stages of podoconiosis were associated with increased severity of depressive symptoms. Podoconiosis patients demonstrated lower median scores across all domains of the WHO QoL Brief in contrast to family members and unaffected neighbors. The burden of depression, anxiety, and stress on podoconiosis patients and their family members is high. Podoconiosis morbidity management programs need to encompass families of patients and integrate continuous mental health support within the broader framework of podoconiosis management.

## Author summary

In this study, we examined the mental health ramifications of podoconiosis, a non-infectious neglected tropical disease caused by prolonged barefoot contact with irritant volcanic soils and affecting poor farming communities in the highlands of Africa, South America, and Asia. Podoconiosis, characterized by lower limb swelling, skin changes, and chronic disability, subjects patients and their families to significant stigma and seclusion. Our comparative cross-sectional study involved podoconiosis patients, their family members, and unaffected neighbors. Using the Depression, Anxiety, and Stress Scale, as well as the WHO Quality of Life Scale—Brief, we assessed each group’s mental health and quality of life status. The results were telling, podoconiosis patients displayed considerably higher odds of severe depressive, anxiety, and stress symptoms while family members had higher anxiety compared to their unaffected neighbors. This stark mental health burden highlights the necessity for comprehensive podoconiosis morbidity management programs, incorporating mental health support for patients and their families.

## Introduction

Podoconiosis is a non-infectious neglected tropical disease (NTD) caused by chronic barefoot contact with irritant volcanic soils in genetically predisposed individuals [1,2]. The condition usually manifests among subsistence farmers in the third decade of life and affects around four million people worldwide, most of whom live in Africa [3,4]. In Rwanda, all districts are endemic to podoconiosis, with nearly 6,000 cases in 2019 [5]. Rwanda has a national podoconiosis prevalence of 68.5 cases per 100,000, varying across districts from 28.3 per 100,000 in Nyarugenge to 307 per 100,000 in Nyamasheke [5].

Podoconiosis typically presents with asymmetric and bilateral swelling of the lower limbs below the knees, as well as nodular skin changes that contribute to disfigurement and chronic disability [3,4]. Patients with podoconiosis frequently experience painful episodes of swelling, inflammation, and fever that impair their social activities and productivity [6]. The clinical progression of podoconiosis can be measured using the Tekola staging system, which has five stages according to the degree of swelling and skin changes. In stage 1, the lower limb edema subsides overnight, with no skin nodules. Stage 2 has edema below the knee that does not resolve, with skin nodules only below the ankles. Stage 3 has edema below the knee that does not resolve, with skin nodules present above the ankles. Stage 4 represents edema above the knee that does not resolve, with skin nodules at any location. In stage 5, the joints are fixated with non-subsiding edema at any part of the leg [7]. Only the early clinical stages of podoconiosis are reversible through treatment [1].

Podoconiosis patients and their families often face internalized, enacted, and perceived stigma, resulting in lower marriage prospects. Those who are married frequently experience verbal and physical domestic abuse [8,9]. Podoconiosis patients are often challenged to contribute to their household income. Altogether, this results in low self-esteem and further social seclusion [9]. Consequently, podoconiosis patients have a poor quality of life (QoL) and mental well-being [10–12]. The mental health consequences of stigma, including depression, anxiety, and emotional distress, contribute to the total illness burden of podoconiosis [13,14]. Podoconiosis patients in Ethiopia, Cameroon, and Rwanda reported high levels of depressive symptoms at 12.6%, 38.5%, and 68.5%, respectively, highlighting a significant mental health burden associated with the disease [11,14,15]. Although not commonly explored, some studies have also shown that the burden of care and extended stigma on family members of podoconiosis patients have mental health implications [8,16].

The disease progression and complications of podoconiosis are managed in podoconiosis morbidity management programs (PMMP). Despite the WHO NTD Roadmap 2030’s emphasis on incorporating mental healthcare within NTD programs, most PMMPs focus on treating physical ailments [10,17,18]. Moreover, there is limited evidence of the extended impacts of podoconiosis on the mental health and QoL of family members of patients, and there are no interventions involving and targeting family members [16,19]. However, the involvement of family members in the management of podoconiosis and lymphatic filariasis has been effective in decreasing disability and increasing QoL, while sub-optimal family dynamics have been shown to make chronic disease care difficult and ineffective [20–22].

In implementing its 2019–2024 NTD strategic plan, Rwanda has rapidly expanded its coverage of podoconiosis management by increasing the number of trained professionals and decentralizing service to health centers [23]. Similarly, Rwanda’s fourth health sector strategic plan has set ambitious objectives for expanding mental health care services through decentralization and integration into primary health care [24]. These two commitments aim to ensure the physical accessibility of both services. However, current PMMPs emphasize managing disease progress and complications with little integration of continuous mental health support to patients and their family members.

This study compared the prevalence and severity of depression, anxiety, and mental stress among podoconiosis patients and their family members with unaffected neighbors who have no personal or family history of podoconiosis. By generating evidence of the extended mental health burden of podoconiosis on family members of patients it sheds light on the cascading yet neglected impact of this disease. It also provides a broader understanding of the larger contribution of podoconiosis to mental health disorders and vice versa in Rwanda. Addressing the mental health consequences of podoconiosis on family members will not only aid the family members but also harmonize family dynamics and possibly contribute to the successful management and elimination of podoconiosis. As Rwanda and other countries expand their PMMP services to rural health centers, this study will assist policymakers and implementers with a critically needed prod to involve family members and integrate mental health services into existing and new PMMPs.

## Methods

### Ethics

Ethical approval for this study was obtained from the University of Global Health Equity (UGHE) Institutional Review Board (UGHE-IRB/2022/057) and the National Health Research Committee (NHRC/2023/PROT/030). Permission to carry out the study was granted by the Rwanda Biomedical Center (RBC) and local district administrations before data collection.

Our data collection procedure considered the vulnerability of podoconiosis patients and their family members. The purpose and process of the data collection were explained to all participants, the voluntary nature of the program was emphasized, and it was made clear that opting to not participate or withdraw would not affect their reimbursement or service at the health center in any way. After explanation, written consent was sought; data was collected only after consent was confirmed. All participants received transportation reimbursement after data collection regardless of finishing the survey.

### Setting

Rwanda is a low-income country in central Africa, and home to 13.2 million people [25]. The country comprises five administrative provinces, which are further subdivided into 30 districts. The Western and Northern provinces are the two most affected by podoconiosis [5]. This study was conducted in Nyamasheke District in Western Province and Musanze District in Northern Province. Nyamasheke and Musanze have podoconiosis prevalence of 119.3 and 86.1 cases per 100,000 people, respectively [5].

Musanze District is the most mountainous district in Rwanda, with multiple dormant volcanoes. More than 50% of Musanze’s and 62% of Nyamasheke’s population live below the poverty line. Agriculture is the primary economic activity for 67.4% of Musanze’s and 73% of Nyamasheke’s inhabitants. Approximately 43.9% of Musanze’s population lives within a 15-minute walk from an improved clean water source, while 43.6% of Nyamasheke’s population lives 30 minutes or more away from improved water sources [26,27]. The mean walking time to a health center is 44 minutes in Musanze and 57.6 minutes in Nyamasheke [28,29].

### Study design

The study employed a survey-based comparative cross-sectional quantitative study design. The 21-question Depression, Anxiety, and Stress Scale (DASS 21), the WHO Quality of Life Scale (WHO-QOL Brief), and the Tekola clinical severity staging system for podoconiosis were used to collect the data [7,30,31].

### Study population

This study targeted all podoconiosis patients aged 18 years and above who lived in the selected districts. The sample size of podoconiosis patients was calculated using the modified Cochran’s formula for small sample populations [32]. We used a confidence level of 0.95, a proportion of 0.68, a margin of error of 0.05, and a total population of 776 podoconiosis patients who are known to live in the study districts [5].


$$n=1+\frac{\frac{n_{0}}{\left(n_{0}-1\right)}}{N}$$

A 1:1:1 ratio was used to determine the sample for family members and unaffected neighbors. Thus, the total sample population was 250 podoconiosis patients, 250 family members, and 250 unaffected neighbors.

Due to the wide and remote geographic distribution of podoconiosis patients, we opted to use health centers as focal points to collect data. As such we randomly selected health centers within the study districts using Excel’s RAND function. All consenting podoconiosis patients within the catchment area of each health center were considered for the study. Initially, ten health centers, five from each Nyamasheke and Musanze district, were randomly selected. Since the sample size was not met with the initial ten, additional health centers were randomly sampled until the sample size was met. In total, 14 health centers, eight from Nyamasheke and six from Musanze, were included in the study.

Family members were purposively selected according to their relationship and residential proximity to podoconiosis patients. Immediate family members (mother, father, son, daughter, husband, wife) who lived in the same household as the patient were primarily selected. In cases where the patient had multiple family members living in the same household, those who were closest by age to the patient were selected. In cases where the patient lived alone, family members, who the patient reported to be closest to them and lived in the same village were selected. Unaffected neighbors were matched to podoconiosis patients by key demographic determinant factors identified in the literature: an age range of ±10 years, same gender, marital status, Ubudehe (socio-economic) category, and educational level were considered a match [33,34]. Family members and unaffected neighbors were grossly evaluated and verbally confirmed to have no physical or mental disabilities.

### Data collection tools

The quantitative survey comprised four sections. Section 1 addressed demographics, including self-declared co-morbidities, participation status in PMMPs, distance from the nearest health facility, and the Ubudehe (socio-economic) category (1-low to 4-high). Section 2 included the DASS 21, with 21 Likert scale questions assessing depression, anxiety, and mental stress levels [30]. Section 3 included six Likert scale questions on perceived stigma towards podoconiosis patients and family members. These questions included questions about the experience of verbal abuse and exclusion from social and religious events. Section 4 included the WHO-QOL Brief with 24 questions of which seven assessed the physical, six assessed the psychological, three assessed the social, and eight assessed the environmental domains of QoL. Each domain was scored individually from 0–100 [31]. Section 5 included the Tekola podoconiosis clinical staging system with five stages (1-mild to 5-severe) [7].

The survey tool was developed in English and translated into Kinyarwanda by three native speakers. It was then back-translated to ensure accuracy before being uploaded to Kobo toolbox for data collection. Four data collectors were trained on the study tools, how to recognize psychological distress, and how to stage podoconiosis using the Tekola staging system. Before data collection, the survey tool was pre-tested on five patients from a PMMP program outside the study districts. Amendments to language and the flow of the data collection procedure were applied according to the feedback.

### Participant recruitment and data collection

The research team obtained permission from the study district administrations to contact local health centers and community health workers (CHWs). CHWs were instructed on the selection criteria and were deployed to recruit all podoconiosis patients within their catchment area. A corresponding family member was selected for each podoconiosis patient recruited. Similarly, an unaffected neighbor (from the same village) with no personal or family history of podoconiosis was matched and selected. CHWs acquired verbal consent from participants and scheduled survey dates. Participants were invited to the local health center for written consent and surveying on the designated survey dates. Surveys were collected in Kobo toolbox on data collector’s phones. After each survey, the podoconiosis patient’s lower limbs were examined by trained data collectors, supervised by an experienced PMMP nurse, using the Tekola Staging system. Participants with high levels of depression or anxiety were referred to a professional mental health assessment at the health center. All data collection activities were conducted in Kinyarwanda.

### Data management and analysis

Data was downloaded from the Kobo toolbox to a password-protected Excel sheet. The data was de-identified using PINs and cleaned on the investigator’s password-protected computer. To facilitate analysis, depression, anxiety, and mental stress scores were summed individually and categorized into Normal, Mild, Moderate, Severe, and Extremely Severe [35]. We combined normal and mild, as well as severe and extremely severe categories due to low counts. Similarly, Ubudehe categories 3 and 4 were combined due to low counts. Tekola podoconiosis stages 1 and 2, as well as 4 and 5, were also merged due to low counts on both extremes. The WHO-QoL Brief results were summed into Physical, Psychological, Social, and Environmental domains and scored from 0–100 [36].

Data was analyzed using SPSS version 27 (IBM). Descriptive analysis was performed for all variables. A Shapiro-Wilk test was used to check for the normality of distribution. Fisher-Exact (X^2^) test was used to analyze the associations between depression, anxiety, and mental stress levels with participant type, demographic variables, as well as Tekola clinical staging scores. The direction and strength of association between depression, anxiety, and mental stress levels and clinical staging scores were determined by a Bonferroni correction and Spearman Rank correlation tests. The association between continuous variables (WHO-QoL Brief, Age, and Distance) with depression, anxiety, and mental stress levels was determined using a Kruskal-Wallis test. For all bivariate statistical tests, *p-values* ≤0.05 were considered significant.

Multicollinearity was assessed using Variance Inflation Factor (VIF) analysis for independent variables with *p* <0.1 in the bivariate tests. VIF values exceeding a threshold of ≥3 were considered indicative of multicollinearity. All QoL domain scores showed a VIF >3; consequently, they were excluded from the final analysis. Independent variables with *p* <0.1 and VIF <3 were included in the multi-ordinal regression model.

## Results

We surveyed 741 participants, 406 (54.8%, Table 1) from Musanze and 335 (45.2%) from Nyamasheke districts, meeting 98.8% of our sample size estimate. Our sample included 246 (33.1%) podoconiosis patients, 247 (33.3%) family members, and 248 (33.5%) unaffected neighbors. Most participants were female (75.2%) and single (35.5%). Almost half (45.3%) had no formal education, of which 52.7% were podoconiosis patients. One-third of our participants (34.1%) were in the lowest Ubudehe category (category 1), and almost three-quarters (71.3%) lived more than one hour from the nearest health center. Most of our participants reported having no family history of mental illness (87.6%).

**Table 1:** Demographics.

Characteristic		Podoconiosis patient (n = 246)	Family member (n = 248)	Unaffected neighbor (n = 247)	Total (N = 741)
		n (%)			
**Sex**	Male	49 (19.9)	83 (33.5)	52 (21.1)	184 (24.8)
	Female	197 (80.1)	165 (66.5)	195 (78.9)	557 (75.2)
**Marital status** ^**a**^	Single	134 (54.7)	87 (35.1)	42 (17)	263 (35.5)
	Married	111 (45.3)	161 (64.9)	205 (83.0)	477 (64.5)
**Education**	None	176 (72.1)	96 (38.9)	62 (25.1)	334 (45.3)
	Primary	58 (23.8)	124 (50.2)	156 (63.2)	338 (45.8)
	≥Secondary	10 (4.1)	27 (10.9)	29 (11.7)	66 (8.9)
**Ubudehe** ^**b**^	Category 1	137 (56.1)	84 (34.4)	35 (14.2)	256 (34.9)
	Category 2	76 (31.1)	116 (47.5)	137 (55.7)	329 (44.8)
	Category 3&4	31 (12.7)	44 (18.0)	74 (30.1)	149 (20.3)
**Self-reported family history of mental illness**	Yes	27 (11.1)	34 (13.7)	27 (11.0)	88 (11.9)
	No	216 (88.9)	214 (86.3)	219 (89.0)	649 (87.6)
**Alcohol use**	I do not drink	110 (44.7)	118 (47.6)	121 (49.2)	349 (47.1)
	1–3 days/week	123 (50.0)	112 (45.2)	114 (46.3)	349 (47.2)
	4–7 days/week	13 (5.3)	18 (7.3)	11 (4.5)	42 (5.7)
**Smoking**	Yes	14 (5.7)	5 (2.0)	9 (3.7)	28 (3.8)
	No	231 (94.3)	243 (98.0)	237 (96.3)	711 (96.0)
**Depression levels**	Normal/mild	50 (20.3)	200 (80.6)	214 (86.6)	464 (62.6)
	Moderate	96 (39.0)	31 (12.5)	24 (9.7)	151 (20.4)
	Severe	100 (40.7)	17 (6.9)	9 (3.6)	126 (17.0)
**Anxiety levels**	Normal/mild	21 (8.5)	125 (50.4)	144 (58.3)	290 (39.1)
	Moderate	61 (24.8)	73 (29.4)	72 (29.1)	206 (27.8)
	Severe	164 (66.7)	50 (20.2)	31 (12.6)	245 (33.1)
**Stress levels**	Normal/mild	118 (48.0)	221 (89.1)	228 (92.3)	567 (76.5)
	Moderate	70 (28.5)	17 (6.9)	13 (5.3)	100 (13.5)
	Severe	58 (23.6)	10 (4.0)	6 (2.4)	74 (10.0)
Median (IQR)					
**Age in years**		55 (23.0)	40 (23.0)	43 (17.0)	45 (23)
**Distance from nearest HC**		90 (75.0)	60 (90.0)	60 (60.0)	60 (80.0)
**WHO QoL Physical Health**		25 (16.0)	57.1 (28.6)	58.3 (25.6)	46.1 (35.7)
**WHO QoL Psychological**		33.3 (20.8)	54.2 (26.0)	58.3 (25.0)	50.0 (33.3)
**WHO QoL Social relationships**		33.3 (25.0)	50.0 (35.4)	62.5 (25.0)	50.0 (41.7)
**WHO QoL Environmental**		28.1 (18.8)	43.8 (25.0)	46.9 (25.0)	40.6 (28.1)

^a^Single contains divorced and widowed participants.

^b^Ubudehe category is a socio-economic classification of Rwandan citizens based on property and income. (Scale: 1 lowest to 4 highest)

The majority (80.5%) of family members were the patients’ immediate family, and 78% lived in the same household as the patient. Nearly three-quarters reported not feeling excluded from religious (74.2%) and social (72.4%) activities because of their family members’ podoconiosis. However, 53.6% reported experiencing verbal abuse from the community due to their family ties with a podoconiosis patient.

Of the 246 podoconiosis patients, 29% were in stage 1, 40.4% in stage 2, 18.8% in stage 3, 4.9% in stage 4 and 6.9% in stage 5. Podoconiosis patients had lower WHO QoL Brief median scores across all domains: physical health (25.0), psychological well-being (33.3), social relationships (33.3), and environmental quality (28.1) when compared to family members and unaffected neighbors. Podoconiosis patients also had more severe levels of depression (40.7%) and anxiety (66.7%). More than one-third of patients reported frequent experiences of verbal abuse from their intimate partners (37.5%), and exclusion from social activities (39%) and religious (33.5%) gatherings. Almost half (48.6%) also felt excluded by their families. Less than half of the patients (39.8%) participated in a PMMP.

There was a significant association (*p* <0.001, Table 2) between depression severity and podoconiosis staging. Bonferroni correction determined a significant difference between severe depression levels and lower Tekola stages (stage 1 and 2) (*p* <0.001), and a Spearman’s rank correlation test revealed a statistically significant, weak positive relationship (Spearman’s rho = 0.22, *p* = 0.001). Podoconiosis staging had no significant association with anxiety levels (*p* = 0.31) or stress severity (*p* = 0.46).

**Table 2:** Association of depression, anxiety, and mental stress levels and Tekola staging.

	Tekola Stage	Stages 1 and 2	Stage 3	Stages 4 and 5	Fisher Exact *p*-value
**Depression levels**	Normal/Mild	37	12	1	<0.001*
	Moderate	78	7	10	
	Severe	55	27	18	
**Anxiety levels**	Normal/Mild	14	6	1	0.31
	Moderate	47	8	5	
	Severe	109	32	23	
**Stress Levels**	Normal/Mild	83	22	12	0.46
	Moderate	52	10	8	
	Severe	35	14	9	

*Significance at p ≤0.05

There was a significant difference (*p* <0.001, Table 3) in all the domains of the WHO QoL scores across the participant categories. Pairwise comparison showed a significant difference (*p* < 0.001) in the QoL of podoconiosis patients from family members and unaffected neighbors in all domains. There was no significant difference between the QoL scores of family members and unaffected neighbors in physical, psychological, and environmental domains. However, there was a significant difference in the social relationship domain (*p* < 0.001).

**Table 3:** Association between WHO-QoL Scores and participant categories.

**WHO-QoL Domains**	**Podoconiosis patient**	**Family member**	**Unaffected neighbor**	**Kruskal-Wallis** ***p*-value**
	Median Score			
**Domain 1: Physical Health**	25 (16.0)	57.1 (28.6)	58.3 (25.6)	0.001*
**Domain 2: Psychological**	33.3 (20.8)	54.2 (26.0)	58.3 (25.0)	0.001*
**Domain 3: Social**	33.3 (25.0)	50.0 (35.4)	62.5 (25.0)	0.001*
**Domain 4: Environmental**	28.1 (18.8)	43.8 (25.0)	46.9 (25.0)	0.001*
**Pairwise comparison**				
**WHO-QoL Domains** **Pairwise comparison**	**Podo patient & Family member**	**Podo patient & Unaffected neighbor**		**Family member & Unaffected neighbor**
**Domain 1: Physical Health**	<0.001*	<0.001*		0.68
**Domain 2: Psychological**	<0.001*	<0.001*		0.24
**Domain 3: Social relationships**	<0.001*	<0.001*		0.001*
**Domain 4: Environmental**	<0.001*	<0.001*		0.35

No participant missing more than 20% data

*Significance at p ≤0.05

Podoconiosis patients have 19.8 higher odds of having severe depression symptoms, 10.7 higher odds of having severe anxiety symptoms, and 13.5 higher odds of having severe stress symptoms compared to their unaffected neighbors (Table 4). Family members of podoconiosis patients had 1.5 higher odds of experiencing higher anxiety symptoms as well. Those who were single and those with no education or only primary education had higher odds of having higher depression levels. Age and distance from the nearest health center were not associated with depression, anxiety, and mental stress levels.

**Table 4:** Adjusted Odds Ratio of depression, anxiety, and mental stress levels and sociodemographic variables.

Characteristics		Depression level			Anxiety level			Stress level		
		*p*-value	Odds ratio	CI 95%	*p*-value	Odds ratio	CI 95%	*p*-value	Odds ratio	CI 95%
**Participant**	Podoconiosis patients	<0.001*	19.8	11.5–33.7	<0.001*	10.7	6.8–16.9	<0.001*	13.5	7.3–24.9
	Family	0.13	1.5	0.9–2.5	0.05*	1.5	1.0–2.1	0.24	1.5	0.8–2.8
	Unaffected	ref								
**Gender**	Male	0.64	0.9	0.6–1.4	0.02*	0.7	0.5–0.9	0.17	0.7	0.4–1.2
	Female	ref								
**Marital**	Single	0.01*	1.8	1.2–2.6	0.16	1.3	0.9–1.8	0.74	1.1	0.7–1.8
	Married	ref								
**Education**	None	0.04*	2.3	1.0–5.3	0.03*	2.0	1.1–3.6	0.17	1.7	0.6–4.3
	Primary	0.04*	2.3	1.0–5.2	0.07	1.7	1.0–3.1	0.06	2.1	0.8–5.4
	≥ secondary	ref								
**Ubudehe** ^**a**^	3 and 4	0.55	1.2	0.7–2.0	0.76	1.1	0.7–1.7	0.79	1.1	0.6–1.9
	2	0.95	1.0	0.7–1.5	0.93	1.0	0.7–1.5	0.71	0.9	0.6–1.5
	1	ref								
**Self-reported family history of mental illness**	Yes	<0.001*	3.0	1.8–5.0	0.001*	2.3	1.4–3.7	<0.001*	2.8	1.6–4.9
	No	ref								
**Age**		0.77	1.0	0.9–1.0	0.36	1.0	0.9–1.0	0.51	1.0	0.9–1.0
**Distance**		0.89	1.0	0.9–1.0	0.06	1.0	1–1.0	0.18	1.0	0.9–1.0

*Statistical significance at p ≤0.05

^**a**^Ubudehe category is a socio-economic classification of Rwandan citizens based on property and income. (Scale: 1 lowest to 4 highest)

## Discussion

The WHO NTD Roadmap 2030 has emphasized the need to incorporate mental health screening into NTD programs; however, the recognition of mental health amongst NTDs with dermatologic manifestations and disability remains low [13,18]. To the best of our knowledge, this is only the second study to compare mental health between podoconiosis patients and unaffected communities, and the first study to include family members in the comparison. Our findings strongly confirm the high burden of depression, anxiety, and mental stress on podoconiosis patients and their family members [13,14,37].

Podoconiosis patients in this study were 19.8 times more likely to experience severe depressive symptoms compared to their unaffected neighbors. Podoconiosis causes a decline in the productivity of patients with up to 90 working days being lost every year [6]. Depression exacerbates this decline in productivity adding to the socioeconomic impact of the disease [8,13,14]. It increases stigma, worsens poverty, and perpetuates a cycle wherein the progression of both physical disability and depressive symptoms are sustained [38,39]. This holds true for most skin-related NTDs such as lymphatic filariasis, cutaneous leishmaniasis, and leprosy where stigma and disability perpetuate poor mental health and vice versa [13,19].

A high prevalence of depressive symptoms has also been reported among podoconiosis patients in Ethiopia (12.6%), Cameroon (38.5%), and Rwanda (68.5%) [11,14,15]. Our findings demonstrated a higher proportion (79.7%) of podoconiosis patients experiencing at least moderate levels of depressive symptoms. The higher prevalences in Rwanda can be explained by the higher rates of mental health conditions in the general population associated with the 1994 Genocide Against the Tutsi. In Rwanda, depression and anxiety disorders are prevalent in 12% and 8.1% of the general population, and 35% and 26.8% of genocide survivors, respectively [40]. Patients in our study had an average age of 55 years, suggesting that they were impressionable youth at the time of the genocide. Nevertheless, we acknowledge various other unexplored sociocultural factors might have contributed to this discrepancy as well.

We considered differences in measurement tools as a possible reason for the higher depression levels in our study compared to the previous Rwandan study. We used the DASS 21 tool while they utilized the PHQ 9 [11]. However, a high correlation between DASS 21 and PHQ-8/9 depression scores has been demonstrated with studies even reporting a lower threshold of PHQ-8 to classify as ‘depression’ than DASS 21 [41,42]. We conclude that while it may still play a role, it is less likely that a difference in sensitivity caused such a significant discrepancy. Alternatively, the predominance of women in our study may have contributed to the higher depression levels. Patients in our study were 80.1% female compared to 69.8% in the previous study [11]. High numbers of female podoconiosis patients have been linked to the lack of agency, low access to education, and protective shoes [43]. This indicates the already vulnerable status of women. The high levels of verbal abuse and stigma faced by female podoconiosis patients put them at higher risks of depression compared to male patients [37,38,44]. Consistently, more than one-third of podoconiosis patients in our study had experienced verbal abuse from their intimate partners due to their condition. Furthermore, women are generally more susceptible to experiencing depression with dermatologic conditions due to the elevated societal expectations about women’s appearance [45]. Regardless, our study augments previous findings, presenting compelling evidence that depression is a highly prevalent comorbidity in individuals affected by podoconiosis.

Single marital status was correlated to severe depressive symptoms. Marriage is an important aspect of the Rwandan social framework. At age 50, only 3% of women and 4% of men in Rwanda will never have been married [46]. This strong social expectation for marriage and the lack of companionship and burden sharing that comes with not being married can expose people to seclusion and stigma [47,48]. Single marital status can also be due to the low marriage prospects of patients and their family members as a result of stigma which is a risk factor for depression [9,49,50].

We also found that low educational status was linked with higher levels of depressive symptoms. The development, progression, and associated disabilities of podoconiosis are linked to low education levels [51]. Conversely, podoconiosis and depression can lower school access, attendance, and completion rates [49,52–54]. This forms a complex dynamic of stressors and risk factors for both depression and podoconiosis such as decreased socioeconomic status, lower prospects of occupation, decreases coping mechanisms, and an overall lower quality of life [55]. Improving the educational status and health education of patients, families, and the community can help tackle the burden of podoconiosis [21,51,56].

We documented a direct correlation between depression levels and advanced stages of podoconiosis, substantiating the positive association between heightened disability and depressive symptoms [57]. This aligns with studies in Ethiopia that reported positive associations between disability, poor productivity, and mental health status in podoconiosis patients [58,59]. A reverse causality of this relationship has also been discussed, suggesting pre-existing mental illness may lead to neglect of podoconiosis, leading to advanced disease [37]. Both assertions demonstrate a cyclical relationship between podoconiosis and mental illness. Patients with severe depression are less able to fully participate in management programs or look after their feet. This would in turn exacerbate deformity, stigma, and the decline in productivity leading to increased depression and anxiety [37,58,60,61].

Podoconiosis patients and their family members experienced significantly higher odds of severe anxiety compared to their healthy neighbors. In contrast, only podoconiosis patients had increased odds of severe mental stress. The disability, verbal abuse as well as societal exclusion that patients experience can cause anxiety, stress, and low QoL [37,61]. High levels of depression, which were identified in our study, are also risk factors for anxiety and stress symptoms [62]. The increased odds of severe anxiety in family members compared to their unaffected neighbors can be attributed to the significant amount of verbal abuse and exclusion they sustain from their community [19,63]. Women, who account for 66.5% of family members in our study, carry a greater responsibility of caring for affected family members due to social norms, and thus, bear an increased burden [64–66]. Caregiving consumes productive time and has been shown to take a toll on the mental health of caregivers [65,67]. A low understanding of the disease due to low educational levels can also predispose family members to increased fear and misconception of the patient’s condition [50]. This would affect the family dynamics and consequently impact the essential emotional and physical support for podoconiosis patients [20,50,58]. Studies on skin-related NTDs, such as leprosy and lymphatic filariasis, support the value of a family’s involvement in disease management [21,68]. Nevertheless, the interconnection between mental illness, QoL, and podoconiosis across patients and their families begs for close monitoring and intervention in PMMPs [10,13]. We did not observe any correlation between anxiety levels and the clinical staging of podoconiosis. This can be attributed to the protracted progress of podoconiosis, which provides a longer duration for psychosocial adaptation to the condition [45].

The overwhelming difference between the depression, anxiety, and stress levels of podoconiosis patients with their family members and unaffected neighbors reflects on the lower QoL of podoconiosis patients in all domains of the WHO QoL [10,12]. The disability associated with the disease affects the productivity, mobility, and overall physical quality of life of patients [69]. Skin-related NTDs, like podoconiosis, are also known to cause shame, self-exclusion, and low self-esteem, which culminate in low social, and psychological QoL [10,70,71]. Family members face social withdrawal, and stigma, leading to poor social QoL, which was demonstrated in this study [19,69]. The role of family relationships is an important factor for both mental illness and NTD progression [21,72]. In this aspect, addressing the QoL and mental health of patients and family members with chronic diseases has been found to lessen the overall impact of chronic conditions [19,73,74].

Our study faced limitations from its cross-sectional design, hindering us from establishing a temporal relationship between contributor and outcome variables. Additionally, our single-point data collection strategy may have decreased the rigor of our results. Other studies have discovered that repeated surveys helped control for over-reporting [14]. While our tool covered several potential risk factors for depression, anxiety, and stress our findings could have been enhanced by incorporating additional factors related to mental health conditions, such as traumatic experiences, fertility, and social support. Due to the limited study population and time constraints, we had challenges adhering to the population matching criteria. To address this, we afforded more time to identify participants that aligned the closest to the matching criteria. The low population size also forced us to collapse various segments of our scores and restricted deeper analysis of the influence of stigma and disability. Although suboptimal, it is unlikely to have influenced our conclusions significantly.

## Conclusion

Our study generated strong evidence illuminating the magnitude of the burden of depression, anxiety, and stress on podoconiosis patients and their family members. The findings underscore the pressing need for integrating mental health services and targeted interventions that extend beyond individuals affected by podoconiosis to encompass the broader familial context. By showcasing the ripple effect of podoconiosis beyond patients, we underscore the importance of community education and awareness about the disease. Ultimately, this study provides compelling evidence for integrating mental health support within the broader framework of podoconiosis management, aiming to enhance the overall quality of life for patients and their families.
